# Effect of Systemic Iron Overload and a Chelation Therapy in a Mouse Model of the Neurodegenerative Disease Hereditary Ferritinopathy

**DOI:** 10.1371/journal.pone.0161341

**Published:** 2016-08-30

**Authors:** Holly J. Garringer, Jose M. Irimia, Wei Li, Charles B. Goodwin, Briana Richine, Anthony Acton, Rebecca J. Chan, Munro Peacock, Barry B. Muhoberac, Bernardino Ghetti, Ruben Vidal

**Affiliations:** 1 Department of Pathology and Laboratory Medicine, Indiana University School of Medicine, Indianapolis, Indiana, 46202, United States of America; 2 Department of Pediatrics, Indiana University School of Medicine, Indianapolis, Indiana, 46202, United States of America; 3 Department of Medicine, Indiana University School of Medicine, Indianapolis, Indiana, 46202, United States of America; 4 Department of Chemistry and Chemical Biology, Indiana University-Purdue University Indianapolis, Indianapolis, Indianam 46202, United States of America; National Institute of Child Health and Human Development, UNITED STATES

## Abstract

Mutations in the *ferritin light chain* (*FTL*) gene cause the neurodegenerative disease neuroferritinopathy or hereditary ferritinopathy (HF). HF is characterized by a severe movement disorder and by the presence of nuclear and cytoplasmic iron-containing ferritin inclusion bodies (IBs) in glia and neurons throughout the central nervous system (CNS) and in tissues of multiple organ systems. Herein, using primary mouse embryonic fibroblasts from a mouse model of HF, we show significant intracellular accumulation of ferritin and an increase in susceptibility to oxidative damage when cells are exposed to iron. Treatment of the cells with the iron chelator deferiprone (DFP) led to a significant improvement in cell viability and a decrease in iron content. *In vivo*, iron overload and DFP treatment of the mouse model had remarkable effects on systemic iron homeostasis and ferritin deposition, without significantly affecting CNS pathology. Our study highlights the role of iron in modulating ferritin aggregation *in vivo* in the disease HF. It also puts emphasis on the potential usefulness of a therapy based on chelators that can target the CNS to remove and redistribute iron and to resolubilize or prevent ferritin aggregation while maintaining normal systemic iron stores.

## Introduction

Neuroferritinopathy or hereditary ferritinopathy (HF) is an autosomal dominant movement disorder caused by mutations in the *ferritin light chain* (*FTL*) gene on chromosome 19q13.3. All mutations occur in exon 4 of the *FTL* gene, leading to the generation of a ferritin light (L) subunit with a longer than normal C-terminal sequence with disordered structure [1]. Clinically, HF presents as a middle-age-onset chorea and dystonia, which may also include extrapyramidal and pyramidal tract signs as well as cerebellar ataxia, dysautonomia, cognitive decline, and psychiatric symptoms. The clinical presentation is highly variable both within and between families, but despite the clinical differences, the neuroimaging is similar in all cases [2, 3]. The main pathologic findings in HF are cystic cavitation of the basal ganglia, the presence of intranuclear and intracytoplasmic ferritin inclusion bodies (IBs) in glial cells and neurons in the central nervous system (CNS), and substantial iron deposition. Intranuclear IBs are the most abundant form of IBs in HF, but intracytoplasmic IBs and significant cytoplasmic ferritin accumulation may also be seen in certain cell types [4]. Mutation carriers may present various systemic diseases before the onset of the CNS disease, but it remains to be determined whether these diseases are associated with the presence of IBs in tissues of multiple organ systems outside the CNS [3]. The presence of IBs in skin and muscle may be useful for the diagnosis of the disease by a biopsy and to monitor the efficacy of therapeutic approaches [4].

Although HF is a rare disease, its study is particularly important due to the presence of a direct link between an abnormality in an iron metabolism protein, the ferritin L subunit, and neurodegeneration. Ferritin consists of 24 subunits (a mixture of L and heavy (H) chains) that can self-assemble into a 480 kDa hollow sphere which can store up to 4500 atoms of iron as a ferrihydrite biomineral. The exterior and interior of the ferritin shell are connected via channels (pores) along symmetry axes at subunit junctions [1]. Analysis of ferritin assembled from L-mutant (Lm) subunits (p.Phe167SerfsX26) has shown remarkable disruption of the 4-fold pores that are formed from four hydrophobically-associated C-terminal E-helices and a reduced ability to store iron, potentially generating reactive oxygen species (ROS) leading to cellular damage [5–8]. *In vitro*, IB formation has been shown to be strongly dependent on iron levels and can be modified by using the iron chelators desferoxamine and phenanthroline [5]. Thus, a therapy aimed at decreasing CNS iron levels toward normal with appropriately designed chelators could reduce pathological iron-induced aggregation and ROS production *in vivo*. However, the use of the iron chelators desferrioxamine and deferiprone (as well as venesection) in patients with HF was reported to cause profound and refractory iron depletion without clinical benefits [2, 9] underlining the lack of an effective treatment for HF.

A mouse model of HF (FTL-Tg) that expresses the mutant Lm p.Phe167SerfsX26 subunit shows a significant decrease in motor performance, shorter life span, misregulation of iron metabolism, and evidence of oxidative damage [10–12]. Ferritin IBs and iron deposition are the main findings in the CNS of FTL-Tg mice, but IBs are also found in organ systems outside the CNS, as in patients with HF [4, 10]. To further understand the role of iron in potentially promoting/accelerating the course of the disease and the use of a chelation therapy aimed at delaying/stopping the progression of HF, we investigated the consequences of iron overload and a chelator treatment in a cell model and the FTL-Tg mouse model of HF.

## Material and Methods

### Ethics statement

This study was carried out in strict accordance with the Guidelines for the Care and Use of Laboratory Animals of the National Institutes of Health. The protocol was approved by the Indiana University School of Medicine Institutional Animal Care and Use Committee (Protocol Number: 10149). All surgeries were performed under anesthesia, and all efforts were made to minimize animal suffering. Mice were anesthetized with acepromazine (2–5 mg/kg) + ketamine (100 mg/kg) given intraperitoneally (i.p.). The animals remained anesthetized during the entire procedure and were euthanized without awakening.

### Animals

Heterozygous FTL-Tg mice expressing a human *FTL* cDNA carrying the *498–499InsTC* mutation in the C57BL/6J background were used. The presence of the transgene was detected by PCR amplification as previously described [10]. Heterozygous FTL-Tg mice were generated by crossing transgenic animals to non-transgenic C57BL/6J mice. Three months-old male and female mice weighing 18–24 g were used in these experiments. Animals were kept under a 12 h–12 h light:dark cycle and allowed free access to food and water.

### Mouse embryonic fibroblasts isolation and culture

Primary mouse embryonic fibroblasts (MEF) were isolated from 13.5 days post-conception mouse embryos from wild-type C57BL/6J and FTL-Tg mice. Embryo carcasses were rinsed in PBS, minced and treated for 15 min in 5 ml of 0.25% trypsin solution (Thermo Scientific Inc., Waltham, MA). Trypsin was then inactivated by adding 25 ml of medium (DMEM with 25 mM glucose, 10% FBS, 100 U/ml Penicillin, 100 μg/ml Streptomycin, 0.25 μg/ml amphotericin B, 6 mM glutamine and 1.5 mM pyruvate; all reagents were from Thermo Scientific Inc.). The cell suspension was passed through a cell strainer and centrifuged for 15 min at 200 g. The cell pellet was re-suspended in fresh media and plated. After 24 h, media was changed again to eliminate dead cells. To obtain immortalized MEF (iMEF), primary cells at passage 2–3 were transfected (FuGENE6, Promega, Madison, WI) with a plasmid containing a SV40 early promotor followed by a retroviral MSV-LTR [13]. Immortalized cells were selected from individual colonies and maintained in media containing 500 μg/ml of the active form of geneticin (G418) (Teknova, Hollister, CA). Cells were kept at 37°C with 5% CO_2_ in a humidified incubator. iMEF from passage 7–16 were used for all the experiments.

### Iron loading and chelator treatment

For cell viability studies, ~10,000 cells/well were plated in a 96 well plate with increasing concentrations of ferric ammonium citrate (FAC) in the presence of 5% fetal bovine serum (FBS) (Thermo Scientific Inc.). Cells were incubated for 3 days and then viability was assessed using the MTT (3-(4,5-dimethylthiazol-2-yl)-2,5-diphenyltetrazolium bromide) (DOT scientific, Inc, Burton, MI) tetrazolium reduction assay. Quantification was performed on a Bio-Tek 880 microplate reader (BioTek, Winooski, VT) at a wavelength of 570 nm with a reference wavelength of 630 nm. For iron accumulation studies, confluent iMEF cultures (>95%) were incubated with 0.5% FBS media in the presence of 100 μM FAC (or PBS as control vehicle) for up to 6 days, changing media daily. Cells were also treated with 100 μM deferiprone (1,2-dimethyl-3-hydroxypyrid-4-one, or L1, or DFP) (Sigma, St. Louis, MO) for up to 3 days in 0.5% serum media with PBS as control vehicle. For hydrogen peroxide toxicity, confluent cells were exposed to 0, 10 or 100 μM FAC in 0.5% serum media for 3 days [14, 15]. Cell viability was assayed after cells were incubated for 1 h in serum-free media and exposed to 1 mM hydrogen peroxide (or PBS as vehicle control) in serum-free media for 24 h.

### In-vivo iron and chelator treatments

For iron treatment, twenty-three FTL-Tg mice (male and female mice were utilized) were randomized to one of the following two groups: (i) iron control (n = 10, equal number of males and females) or (ii) chronic iron loading (n = 13, 7 males and 6 females). Control FTL-Tg mice received placebo treatment with normal saline (0.5 mL/mouse/day) by i.p. injection once per week for a period of 4 weeks. FTL-Tg mice in the chronic iron overload group received one injection of iron dextran (100 mg/kg i.p./mouse) (Sigma) per week for a period of 4 weeks. The dose of iron dextran administered to these mice was based on previous investigations [16, 17]. Body-weights were measured weekly for all groups. One month was allowed for equilibration of iron after overloading, after which the animals were analyzed.

For DFP treatments, thirty-six FTL-Tg mice were randomized to one of the following treatment groups: (i) chelator control (n = 11, 6 males and 5 females); (ii) chelator low-dose (50 mg/Kg/day; DFP_50_) (n = 11, 6 males and 5 females), or (iii) chelator high-dose (100 mg/Kg/day; DFP_100_) (n = 14, 7 males and 7 females). Control mice received placebo treatment with normal saline (0.5 mL/mouse/day) by i.p. injection. DFP was administered systemically by i.p. route. Mice received a total of 70 doses, 5 out of 7 days per week for a period of 14 weeks. Mice were observed immediately before dosing each day and again for at least 15 minutes afterwards. The dose of DFP administered to these mice was based on previous investigations [16, 17]. Body-weights were measured weekly for all groups, and mice were analyzed at the end of the treatment.

### Serum biochemistry and hematological analyses

Blood samples were obtained prior to perfusion by cardiac puncture. Serum was separated by centrifugation and used to determine unsaturated iron binding capacity (UIBC) and iron using a COBAS MIRA Plus Chemistry Analyzer (Roche Diagnostics, Indianapolis, IN). A complete blood cell count (CBC) was performed on whole blood using a Mascot HemaVet950FS (Drew Scientific, Miami Lakes, FL) automated processor as previously described [18].

### Histology and immunohistochemistry

After anesthesia, mice were transcardially perfused with 0.9% saline and then brains and organs were fixed by immersion in 4% paraformaldehyde solution for 24 h at 4°C, embedded in paraffin and sectioned. Eight-micrometer-thick sections were stained by the Hematoxylin-Eosin (H&E) method. In addition, Perls’ method for ferric iron enhanced with DAB was used as previously described [10]. Immunohistochemical labeling was also carried out following published protocols [10, 11]. For immunohistochemistry, sections were incubated overnight at 4°C with the primary antibodies (Abs) in blocking solution. We used primary Abs raised against mutant L (Lm; Ab1283) [4] and heavy chain (H) (Ab65080; Abcam, Cambridge, MA). Immunostaining was visualized using the avidin-biotin system (Vectastain; Vector Laboratories, Burlingame, CA) and 3,3’-diaminobenzidine (Sigma) as the chromogen. The sections were counterstained with cresyl violet or H&E, and images were captured by a digital camera coupled to a Leica DM4000B microscope (Leica Microsystems, Buffalo Grove, IL).

### Western blot analysis

Cellular or tissue fractions were prepared using the CelLytic NuCLEAR Extraction Kit (Sigma) following the manufacturer’s procedures. After cells were incubated in the lysis buffer, the supernatant (containing soluble cytoplasmic proteins) was separated by centrifugation and considered the “supernatant” fraction. The pellet, containing nuclei, cell membranes, and insoluble proteins was analyzed as the “pellet” fraction. The purity of the fractions was assessed by immunoblotting with antibodies against the nuclear protein Histone H2A and the cytosolic protein GAPDH. Actin distributed consistently between the supernatant and pellet. Protein extracts were aliquoted and stored at -80°C until used. Protein concentration was determined by using a protein assay dye reagent kit (Bio-Rad, Hercules, CA). Between 3–4 μg of cell or 2.5–10 μg of tissue protein lysates were run in denaturing 16% acrylamide Tris-Tricine gels and transferred to Protran nitrocellulose membranes (GE Healthcare, Pittsburgh, PA). Membranes were blocked for 1 h in 4% low fat dried milk in TBS containing 0.1% Tween-20 (TBS-T) and then incubated for 16 h with the primary Ab. The following Abs were used: Ab1283, anti-L (Ab63010; GeneTex, Irvine, CA), anti-H, and anti-β actin (Sigma). After washing in TBS-T, the membranes were incubated with peroxidase-conjugated secondary Ab (Cayman Chemical, Ann Arbor, MI) (1:5,000) for 1 h. Membranes were developed using the ECL chemiluminescent detection system (GE Healthcare). Equal protein load was confirmed using anti-β-actin Abs. The films were scanned and the densities of the bands measured using NIH ImageJ Software. The densities of the bands were normalized against those of β-actin and the mean ratios calculated. Statistical analysis was performed using GraphPad Prism (GraphPad Software, La Jolla, CA).

### Non-heme iron

Iron was determined in cell homogenates and tissue homogenates from the liver and kidney. iMEFs were trypsinized, pelleted in DMEM supplemented with 10% FBS, rinsed 3 times in PBS and digested for 2 hours at 55°C rocking in 50 mM NaOH. Cellular fractions from tissue samples were prepared using the CelLytic NuCLEAR Extraction Kit. Non-heme iron content was determined spectrophotometrically by the ferrozine method. Briefly, samples (100 μl) were incubated for 2 hours at 65°C with 1 volume of iron releasing reagent (2.25% KMn_4_ and 0.7 N HCl) and 1 volume of 10 mM HCl to ensure all the protein-complexed iron is effectively released. Thirty μl of ferrozine solution [6.5 mM 3-(2-pyridyl)-5,6-bis(4-phenylsulphonic acid)-1,2,4-triazene, 6.5 mM neocuprine, 2.5 M ammonium acetate, 1 M ascorbic acid] (Sigma) was added to the cooled reaction, and the absorbance was determined at 570 nm. To calculate the absolute iron content, a standard curve of ferric ammonium citrate solution of known concentration was used.

### RNA isolation and multiplex expression analysis

Mice were anesthetized, transcardially perfused with 0.9% saline, and the brain and liver removed. Microdissected cerebral cortex and liver samples were placed in 500μl of RNA later (Qiagen, Valencia, CA) and frozen at -20°C. RNA was isolated using RNeasy Lipid Tissue Mini Kit (Qiagen) according to the manufacturer’s protocol. Samples were treated on column with the RNase free DNase Kit (Qiagen) according to the manufacturer instructions. Reverse transcription was performed on 25 ng of total RNA for each sample followed by multiplex PCR, and fragment separation by capillary electrophoresis using the GeXP Chemistry Protocol (Beckman Coulter, Indianapolis IN). Gene specific primer pairs (without universal tags) used in RT-PCR were as described [11, 18]. Fragments were separated using a CEQ 8000 Automated Capillary DNA sequencer/Genetic Analysis Systems (Beckman Coulter), and analyzed with the GenomeLab GeXP Genetic Analysis System (Beckman Coulter) using the following fragment analysis parameters: slope threshold = 0.9999, peak height threshold = 800 rfu, peak size < 375, peak size > 150, dye = D4. Multiplex-specific fragments were selected by applying exclusion filters and the data exported to eXpress Analysis software, where they were normalized against the mouse *polymerase II polypeptide A* (*Polr2a*) gene or the *β-actin* gene as described [11, 18]. Relative mRNA level values for each of the triplicates for each sample were averaged and the mean for the replicates were compared between treated and control FTL-Tg mice by an unpaired two-tailed t-test using GraphPad Prism. Differences in relative mRNA levels with p-values < 0.05 were considered statistically significant. Data are reported as mean ± standard error of the mean (SEM).

### Statistics

Data is presented as mean ± SEM. IC_50_ was calculated fitting the viability curve to a four parameter logistic equation. For each parameter, normality was tested by the Shaphiro-Wilk test. We used One-Way Analysis of Variance, followed by the LSD post-hoc test, when comparing data with more than 2 groups. Two-Way Analysis of Variance was used when comparing samples with 2 independent factors. When only 2 sample groups were compared, we performed an unpaired two-tailed t-test using GraphPad Prism. A value of p<0.05 was considered statistically significant.

## Results

### Iron and ferritin accumulation in iMEFs. Role of DFP in iron accumulation and cell survival

**Fig 1:**
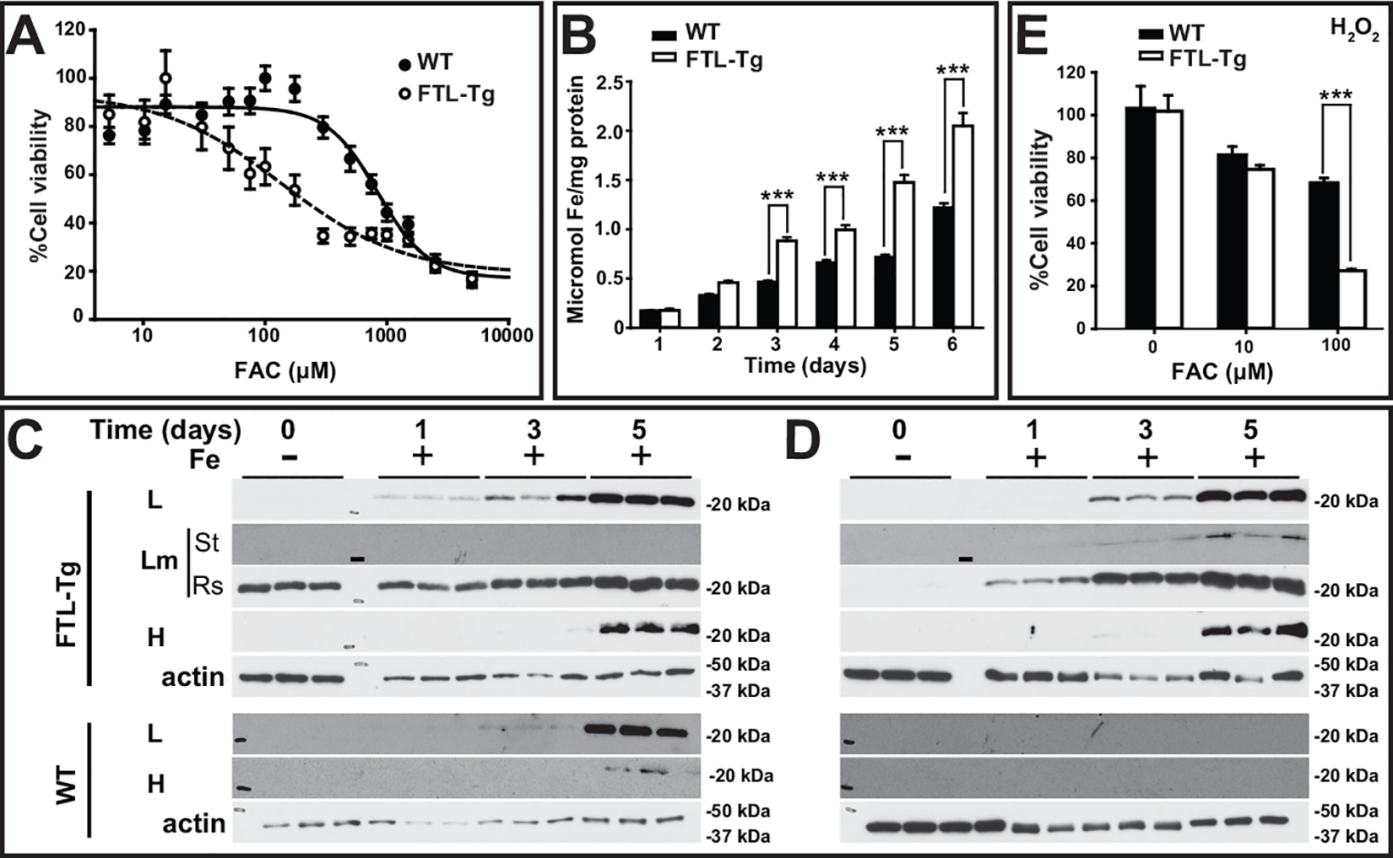
Iron loading and response to oxidative stress of iMEFs. Compare to non-transgenic wild-type (WT) control cells, iMEFs from FTL-Tg mice show a decreased viability in the presence of increasing concentrations of iron (A). iMEFs from FTL-Tg mice accumulated a significantly higher level of intracellular iron after 3 days of iron (100 μM) loading (p < 0.001). Cells were iron-loaded for up to 6 days (B). The levels of wild type L, Lm, and H polypeptides were determined in the supernatant (C) and the pellet (D) after cells were exposed for 1, 3 or 5 days to iron as in B. As control (0 days), cells were cultured for 3 days without iron. Lm was detected in the resolving (Rs) gel above the 20 KDa marker and also in the stacking (St) gel (the line represents the 250KDa marker). The blots show triplicates from a representative experiment. A significant (p < 0.001) decreased in cell viability was observed in confluent cells from FTL-Tg mice cultured in the presence of FAC after being exposed to hydrogen peroxide. % of cell viability was calculated as the viability of cells with PBS in the same iron load context (E). All experiments were repeated a minimum of three times to ensure reproducibility.

**Fig 2:**
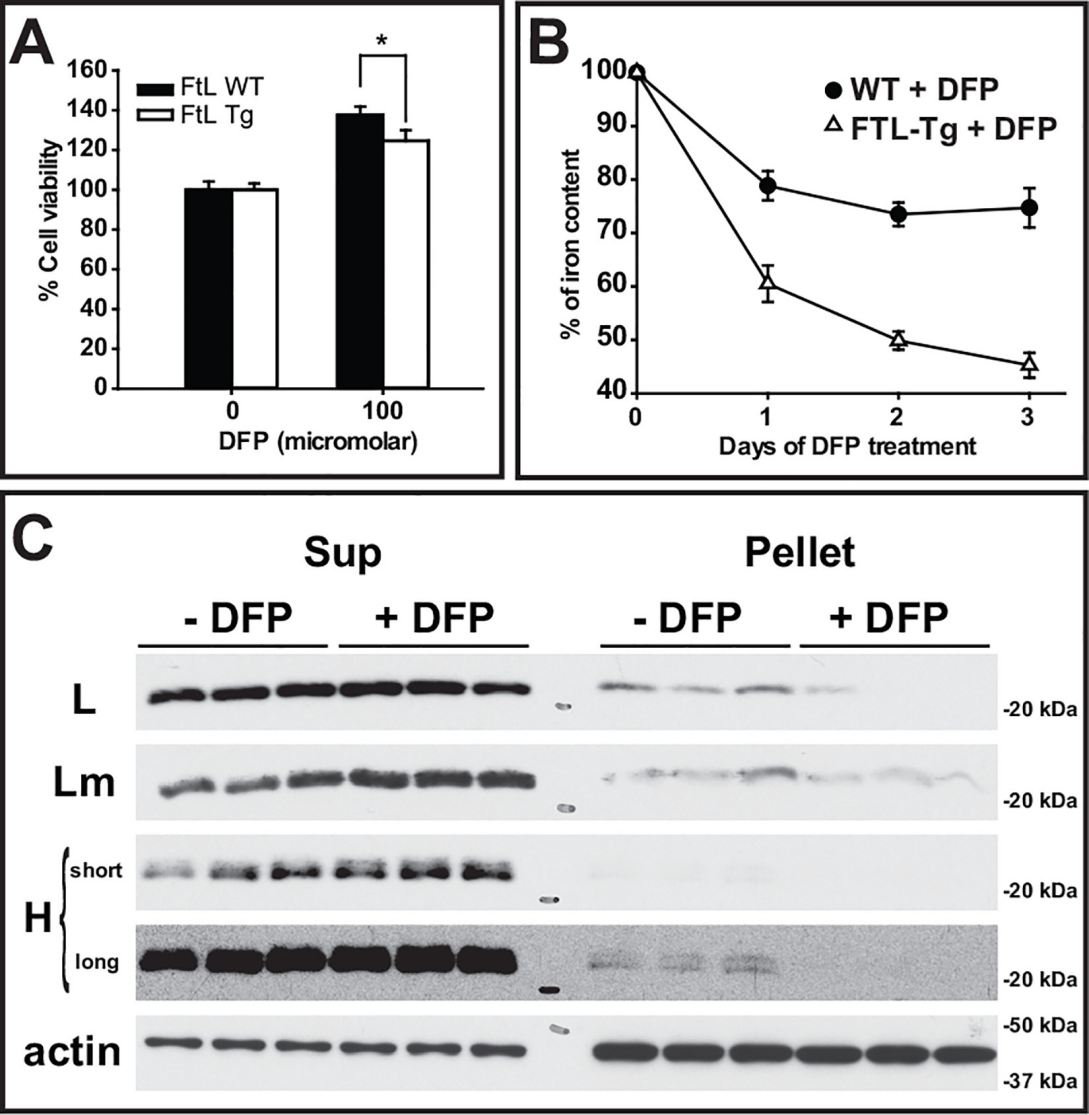
DFP treatment of iMEFs. Cell viability of iron loaded iMEFs (3 days with 100 μM iron) increased considerably after 3 days of DFP exposure (A). The iron content of the cells decreased significantly after DFP treatment, in particular in iMEFs from FTL-Tg mice. Values are expressed as % of iron content of cells treated with DFP compared to cells treated with PBS for the same period of time (B). The levels of wild type L, Lm, and H polypeptides were determined in the supernatant (Sup) and pellet after cells were exposed for 3 days to DFP or PBS (-DFP) (C). For H, long and short exposure times are shown. The blots show triplicates from a representative experiment. All experiments were repeated a minimum of three times to ensure reproducibility.

Viability studies show that iMEFs from FTL-Tg mice were more sensitive to iron than iMEFs from C57BL/6J wild-type (WT) control mice (Fig 1A), with a lower IC_50_ (124μM) than control cells (844μM). Compared to control cells, iMEFs from FTL-Tg mice begin to accumulate significantly more iron after 3 days of incubation in the presence of 100 μM FAC (*p* < 0.001) (Fig 1B). Western blot analysis shows accumulation of ferritin in both, the supernatant (Fig 1C) and pellet (Fig 1D) with iron loading. Interestingly, Lm was also detected in the stacking gel (St) of iron-loaded iMEFs from FTL-Tg mice, suggesting the formation of SDS-resistant aggregates, as we previously described [10]. Addition of hydrogen peroxide to iron loaded cells led to a significant (*p* < 0.001) decrease in the viability of iMEFs from FTL-Tg mice compared to iMEFs from wild-type control mice (Fig 1E). Treatment of the iron loaded cells with DFP led to a significant increase (~30%) in cell viability in iMEFs from control (*p* < 0.001) and FTL-Tg mice (*p* < 0.001), but the effect was more significant in the control cells (*p* = 0.042) (Fig 2A). A significant decrease in iron content (Fig 2B) was also observed, which was more significant in iMEFs from FTL-Tg mice (*p* < 0.001) with a redistribution of ferritin proteins from the pellet to the supernatant (Fig 2C). The difference observed in iron accumulation in response to the chelator treatment suggests that the iron accumulating in iMEFs from FTL-Tg mice may be loosely bound to ferritin, highlighting the role of intracellular iron as a major modulator of ferritin aggregation *in vivo* and the potential use of a chelator-based therapy for the disease.

### Iron overload and systemic iron homeostasis in FTL-Tg mice

**Fig 3:**
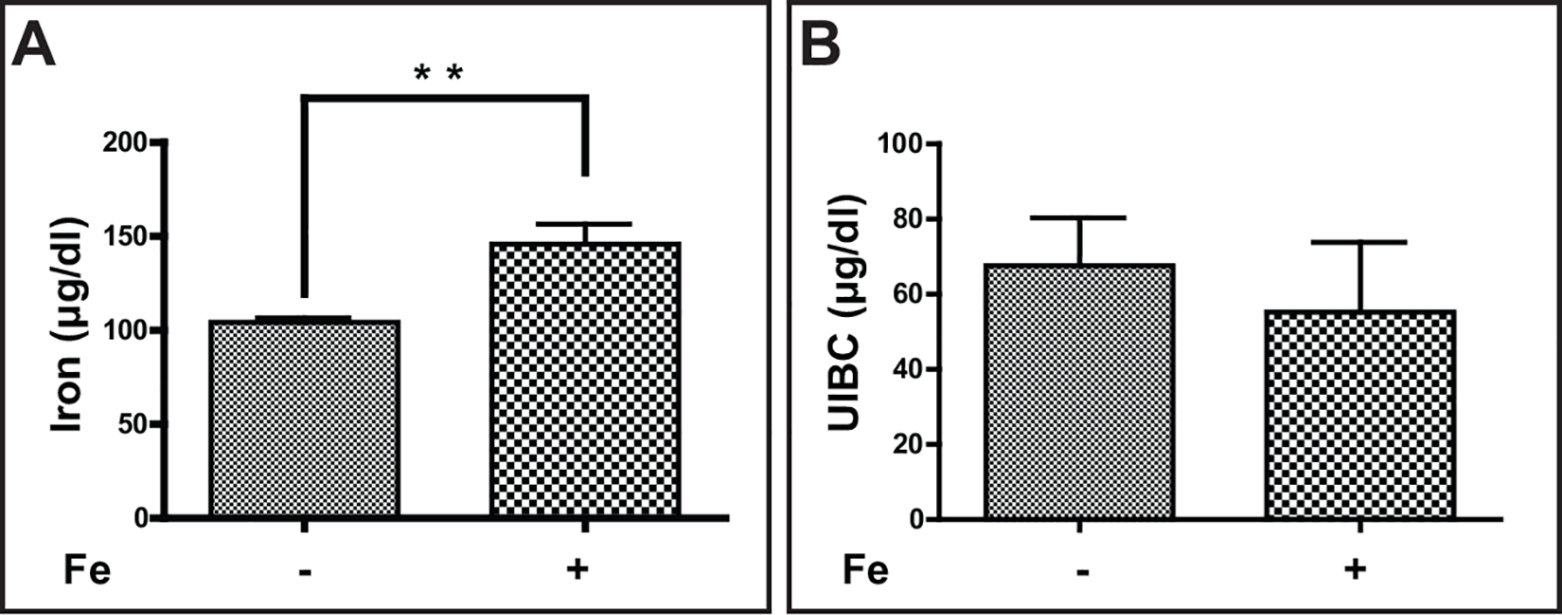
Serum iron levels and UIBC levels in iron-treated mice. Serum iron levels were significantly (p < 0.01) increased in iron treated mice (145.9 ± 10.6 μg/dl) compared to untreated mice (104.3 ± 2.2 μg/dl) (A). A decrease of UIBC levels in iron treated mice was observed but did not reach statistical significance (p = 0.6055) (B). For serum studies, FTL-Tg untreated (control; n = 10, 5 males and 5 females) and treated (n = 12, 6 males and 6 females) mice were analyzed. Samples were analyzed by a two-tailed t-test and results considered significant for p < 0.05.

No significant difference in body weight between iron dextran-treated and control FTL-Tg mice was observed (not shown). No mortality was observed due to the treatment. To assess the impact of iron loading on the major systemic iron utilization pathway, we determined serum iron levels, unsaturated iron binding capacity (UIBC), and hematological parameters. Compared to untreated mice, a significant (*p* = 0.0022) increase in serum iron levels was observed in iron loaded mice (Fig 3A). UIBC levels were lower in iron loaded mice, but this change did not reach statistical significance (*p* = 0.6055) (Fig 3B). No significant differences were observed in red cell (RBC) and leukocyte (WBC) counts, serum hemoglobin (Hb) values, and mean corpuscular hemoglobin concentration (MCHC) between controls and iron loaded FTL-Tg mice. A significant elevation of the values of hematocrit (HtC; *p* = 0.045), mean corpuscular volume (MCV; *p* < 0.001), mean corpuscular hemoglobin (MCH; *p* = 0.003), red cell distribution width (RDW; *p* = 0.0012), platelets (Pt; *p* = 0.003), and mean platelet volume (MPV; *p* = 0.0021) was observed in the iron loaded group (Table 1).

**Table 1:** Hematological parameters of FTL-Tg mice treated with iron dextran (Fe) (n = 13) compared to age-matched FTL-Tg untreated (Control (no Fe)) (n = 10). DFP-treated FTL-Tg mice at a low dose (DFP_50_) (n = 11) or a high dose (DFP_100_) (n = 14) were compared to age-matched FTL-Tg untreated (Control (no DFP)) (*n =* 11). The following hematological parameters were measured: RBC, red blood cells number (x 10^6^/ml); WBC, white blood cells (x 10^3^/ml); Hb, hemoglobin (g/dl); HtC, hematocrit (%), mean corpuscular volume (MCV), mean corpuscular hemoglobin (MCH), mean corpuscular hemoglobin concentration (MCHC), red cell distribution width (RDW), Platelet (Pt), and mean platelet volume (MPV). Significant differences compared to controls (p < 0.05) are indicated by *. Values are mean ± SEM.

*FTL-Tg*	RBC	WBC	Hb	HtC	MCV	MCH	MCHC	RDW	Pt	MPV
***No Fe***	8.2 ± 0.3	4.8 ± 0.5	10.8 ± 0.4	36.2 ± 1.4	44.0 ± 0.2	13.1 ± 0.2	29.7 ± 0.3	16.5 ± 0.1	380 ± 19	4.06 ± 0.05
***Fe***	8.5 ± 0.3	4.9 ± 0.5	11.9 ± 0.3	40.4 ± 1.3*	47.6 ± 0.5****	14.0 ± 0.1***	29.42 ± 0.3	17.76 ± 0.3**	541 ± 28***	4.33 ± 0.05**
***No DFP***	8.7 ± 0.7	4.1 ± 0.5	11.9 ± 0.9	38.6 ± 2.9	44.6 ± 0.7	13.7 ± 0.2	30.7 ± 0.3	17.8 ± 0.5	447 ± 32	4.25 ± 0.08
***DFP***_***50***_	8.8 ± 0.4	4.9 ± 0.7	12.1 ± 0.6	38.6 ± 1.8	44.1 ± 0.2	13.8 ± 0.1	31.4 ± 0.2	17.1 ± 0.2	483 ± 19	4.32 ± 0.05
***DFP***_***100***_	7.5 ± 0.5	4.1 ± 0.5	11.0 ± 0.7	36.0 ± 2.2	47.9 ± 0.6**	14.7 ± 0.2***	30.6 ± 0.3	18.5 ± 0.3	476 ± 24	4.87 ± 0.16**

**Fig 4:**
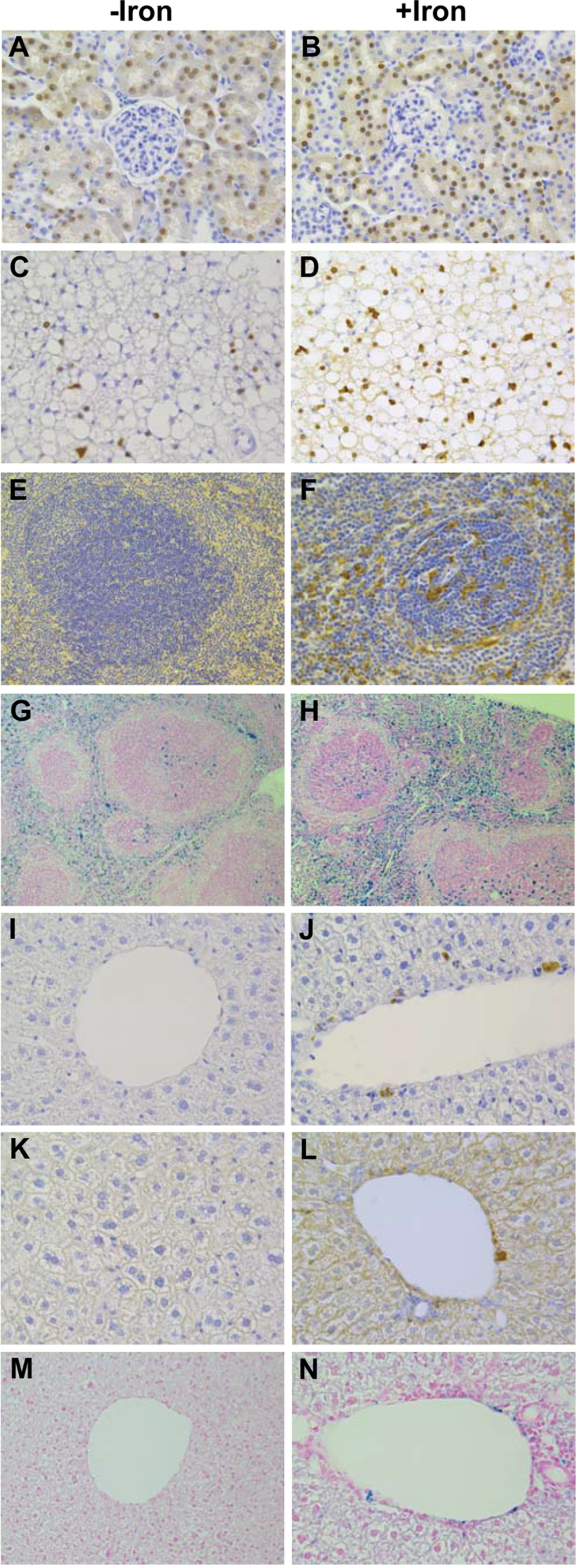
Histological and immunohistochemical studies of iron-loaded and control FTL-Tg mice. Analysis of paraffin embedded sections from FTL-Tg control (A, C, E, G, I, K, M) and iron-loaded (B, D, F, H, J, L, N) mice. Sections shown are from kidney (A, B), adipose tissue (C, D), spleen (E-H), and liver (I-N). Sections were immunostained with Abs against the mutant L chain (A-D, I, J) and against the H chain (E, F, K, L), and stained with the Perls’ Prussian blue method (G, H, M, N). Original magnifications x10 (G, H), x20 (E), x40 (A-D, F, H-N).

Pathologic analysis was performed on tissue sections from the heart, muscle, liver, stomach, intestine, spleen, adipose tissue, lungs, and reproductive organs (testis and ovaries). To be able to detect small changes in ferritin and iron deposition, we analyzed young heterozygous FTL-Tg mice, before high levels of ferritin were deposited. Iron treatment did not seem to modify the tissue distribution of ferritin IBs in FTL-Tg mice, although tissues from iron loaded mice appeared to have a larger number of IBs (Fig 4A–4D). No major differences were observed during the pathologic examination of the organs; however, iron-containing ferritin aggregates in the spleen and liver were remarkable different in iron loaded FTL-Tg mice (Fig 4E–4N). Ferritin aggregates in the liver were observed only in the iron-loaded group and appeared morphologically different from the intracellular IBs characteristic of HF [10, 19] (Fig 4I–4N) in agreement with previous work on iron overload in mice [20, 21]. Aggregates were stained by Abs against the Lm and H chains. Anti-H also showed a significant intracellular accumulation of the H chain in the liver parenchyma, including hepatocytes (Fig 4L). Liver ferritin aggregates were not detected in FTL-Tg control mice. At 3 month of age, heterozygous transgenic mice do not show ferritin IBs in the liver since the expression of the transgene in the liver is very weak (Fig 4I–4K), but a few ferritin IBs containing the mutant subunit may be found in the liver of homozygous mice of the same age [10]. Liver ferritin aggregates were also strongly stained by the Perls’ method for ferric iron (Fig 4M and 4N).

**Fig 5:**
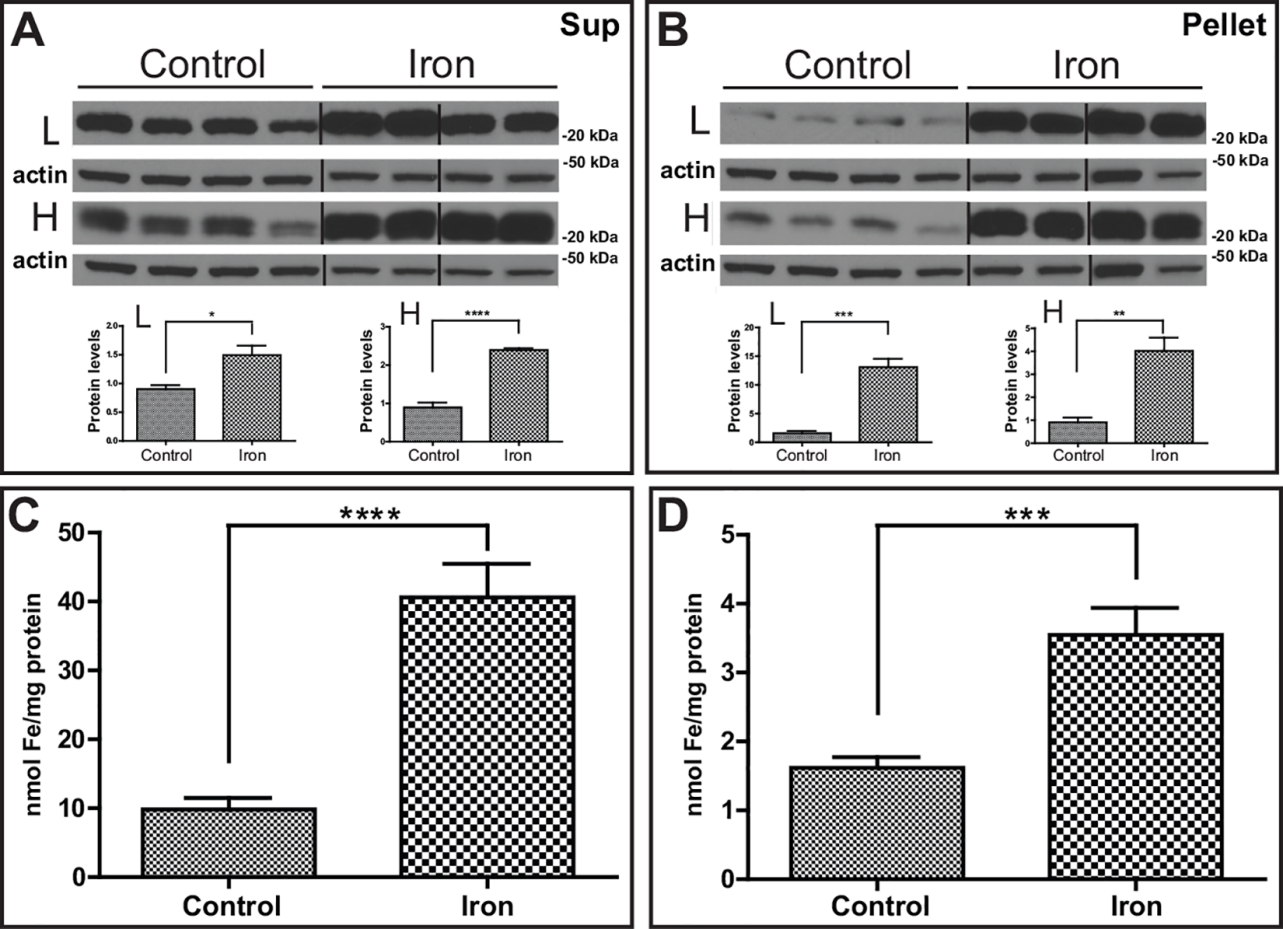
Western blot analysis and non-heme iron of liver of iron-loaded and control FTL-Tg mice. The levels of wild type L and H polypeptides in the liver in the supernatant (A) and the pellet (B) were determined by western blot. β-actin was used as loading control. The vertical lines in the panels denote non-adjacent bands from the same blot. Representative blots are shown for four male mice on each group. Densitometric analysis from three independent experiments shows a statistical significant difference between the controls and iron-loaded mice (*p < 0.05). By the colorimetric ferrozine method, a significant increase in the levels of non-heme iron in the liver of iron-treated FTL-Tg mice compared with non-treated FTL-Tg controls was observed in the supernatant (p < 0.0001) (C) and the pellet (p = 0.0004) (D).

To quantitatively assess protein deposition in the liver, we performed western blot analysis. We observed that the levels of the L and H subunits were significantly increased in iron loaded FTL-Tg mice in both supernatant (L, p = 0.0165; H, p < 0.0001) and pellet(L, p = 0.0003; H, p = 0.0023) (Fig 5A and 5B). The Lm subunit was not detected in the liver samples. In addition, we observed a statistically significant increase (p < 0.0001) in the levels of non-heme iron in the same protein fractions of iron-treated FTL-Tg mice compared with non-treated FTL-Tg controls as determined by the colorimetric ferrozine method. A difference of over 300% was observed between control FTL-Tg mice (9. 97 ± 1.69 nmol Fe/mg protein) and iron-treated FTL-Tg mice (40.60 ± 4.86 nmol Fe/mg protein) in the supernatant (Fig 5C). Similarly, a significant increase (p = 0.0004) was also observed in the pellet of the iron loaded group (Fig 5D). Analysis by multiplex RT-PCR of a total of 18 genes that play a role in iron metabolism and related pathways in the liver was performed in triplicate. *mRNA* levels were compared between untreated (control) and iron loaded FTL-Tg mice. A significant increase in the levels of *Ftl* (*p* < 0.0001), *Fth1* (*p* < 0.05), *hepcidin* (*Hamp)* (p < 0.001), *AcoI* (*p* < 0.05), *Sod1* (p < 0.05), and *Hmox1* (p < 0.05) *mRNA* was observed. A significant decrease in the levels of *Tfrc* (p < 0.01) was also observed (S1 Fig). An increase in the levels of *Cp* was observed, but it did not reach statistical significance (p = 0.0526). No other significant differences were observed in the expression of the genes analyzed in the liver plex.

### Iron overload and brain iron homeostasis in FTL-Tg mice

**Fig 6:**
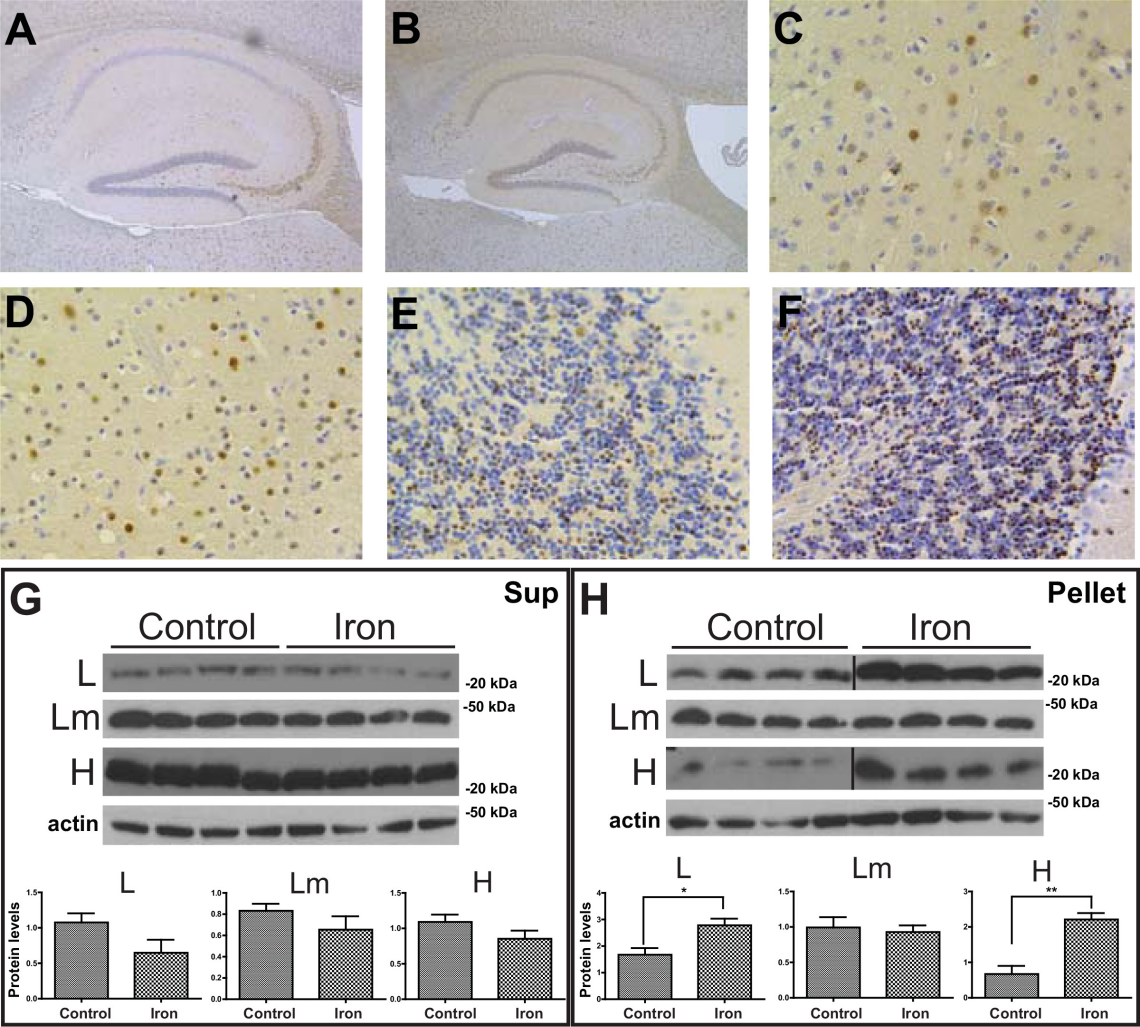
Neuropathological examination and western blot analysis of iron-loaded and control FTL-Tg mice. Analysis of paraffin embedded sections from control (A, C, E) and iron-loaded (B, D, F) FTL-Tg mice shows the presence of ferritin IBs. Sections shown are from the hippocampus (A, B), striatum (C, D), and cerebellum (E-F). Sections were immunostained with Abs against the mutant L chain (A-F). Original magnifications x5 (A, B), x40 (C-F). The levels of wild type L, L mutant (Lm), and H polypeptides in the cerebral cortex in the supernatant (G) and the pellet (H) were determined by western blot. β-actin was used as loading control. The vertical lines in panel H denote non-adjacent bands from the same blot. Representative blots are shown for four male mice on each group. Densitometric analysis from three independent experiments showed a statistical significant difference between the controls and iron-loaded mice in the levels of the L and H subunit in the nuclear-insoluble fraction (H). (*p < 0.05).

To assess the impact of iron overload in ferritin deposition in the CNS of FTL-Tg mice, we analyzed brains of mice injected with iron dextran or normal saline as describe above. Neuropathologic examination of brain tissues showed the presence of IBs throughout the brain as previously reported in FTL-Tg mice [10], but did not reveal major histological differences between control and iron loaded FTL-Tg mice (Fig 6). To quantitatively assess protein deposition in the cerebral cortex, we performed western blot analysis. The levels of the different subunits were not significantly different between control and iron loaded FTL-Tg mice in the supernatant (Fig 6G); however, a significant increase in the levels of the L and H subunit was observed in the pellet (L, p = 0.0205; H, p = 0.0019), without significant changes in the levels of the mutant L chain (Lm) (Fig 6H). Analysis by multiplex RT-PCR showed a significant decrease in the levels of *Tfrc mRNA* (p < 0.05) in iron-loaded FTL-Tg mice; however, this was the only significant change in gene expression detected in the cerebral cortex. Analysis of the expression of the ferritin transgene by multiplex PCR did not reveal any significant differences in the expression of the transgene in the brain between controls and iron-loaded FTL-Tg mice (not shown).

### Effects of the iron chelator DFP in systemic iron homeostasis of FTL-Tg mice

No significant differences in body weight between DFP-treated and control FTL-Tg mice were observed after the 14-week treatment (not shown), and there was no mortality associated with DFP treatment. A week after the last injection, blood and tissues were analyzed. At necropsy, a remarkable finding was a significant atrophy of the testis in males receiving a dose of 100 mg/kg/day (S2 Fig). To assess the impact of the iron chelation treatment on the major systemic iron utilization pathway, we determined serum iron levels, UIBC, and hematological parameters. No significant differences were observed in serum iron and UIBC levels between controls and the DFP_50_ treated group (not shown). In addition, treatment with DFP at a low dose did not lead to significant differences in hematological parameters between treated mice and controls. Compared to controls, treatment with the high dose of DFP did not change significantly red cell counts, leukocyte counts, serum hemoglobin values, hematocrit, MCHC, and Pt values; however, significant differences were observed in the values of MCV (p < 0.0001), MCH (p < 0.0001), RDW (p = 0.0394), and MPV (p = 0.007) (Table 1).

**Fig 7:**
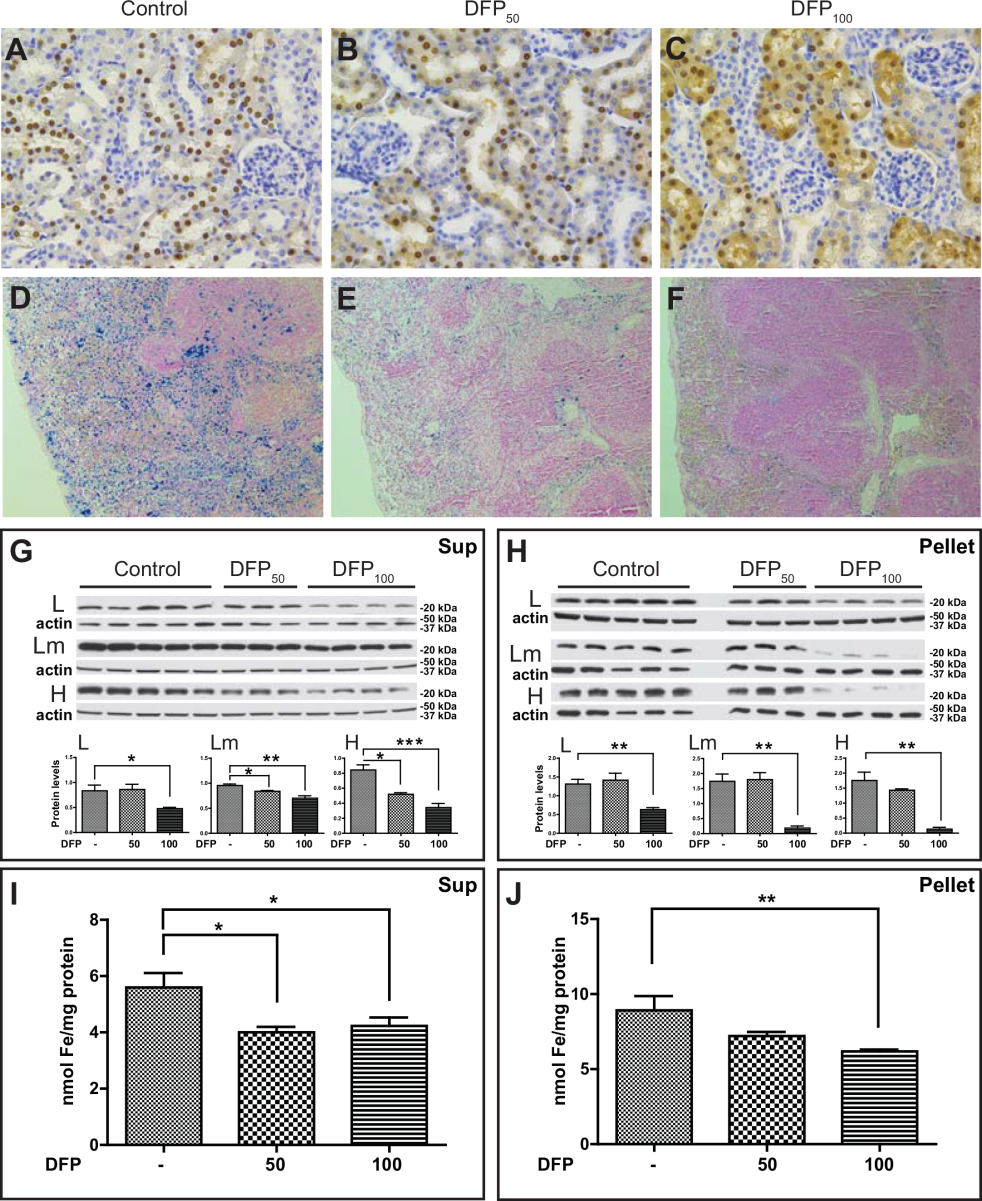
Ferritin and iron deposition in the kidney and spleen of DFP-treated mice. Histological and immunohistochemical studies of DFP-treated and control FTL-Tg mice (A-F). Analysis of paraffin embedded sections from control (A, D), DFP_50_ (B, E), and DFP_100_ (C, F) treated FTL-Tg mice. Sections shown are from kidney (A-C) and spleen (D-F). Sections were immunostained with Abs against the mutant L chain (A-C) or stained with the Perls’ Prussian blue method (D-F). Original magnifications x10 (D-F), x40 (A-C). Western blot analysis of protein samples from the kidney using antibodies specific for the L, Lm, and H chains (B). β-actin was used as loading control. Representative blots are shown for five control, three DFP_50_, and four DFP_100_ male mice. Densitometric analysis from three independent experiments shows statistical significant differences between the controls and DFP-treated mice in the supernatant (G) and the pellet (H). (*p < 0.05). By the colorimetric ferrozine method, a decrease in the levels of non-heme iron in the kidney of DFP-treated FTL-Tg mice compared with non-treated FTL-Tg controls was observed in the supernatant (DFP_50_, p = 0.0190; DFP_100_, p = 0.0299) (I) and the pellet (DFP_100_, p = 0.0032) (J).

**Fig 8:**
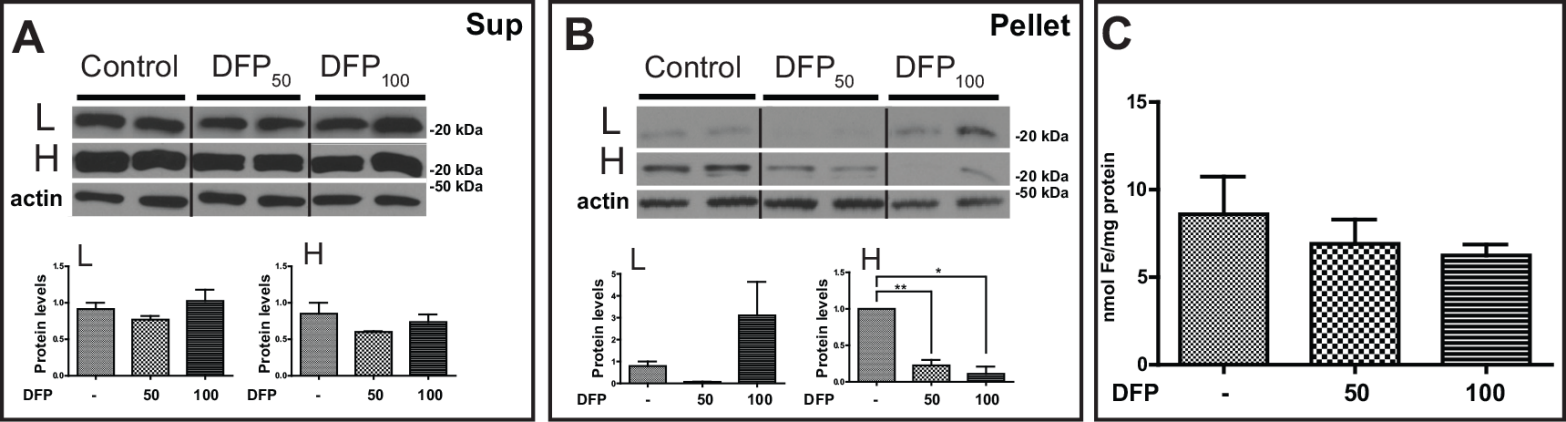
Western blot analysis and non-heme iron of liver of DFP-treated and control FTL-Tg mice. The levels of wild type L and H polypeptides in the liver in the supernatant (A) and the pellet (B) were determined by western blot. β-actin was used as loading control. The vertical lines denote non-adjacent bands from the same blot. Representative blots are shown for two male mice of each group. Densitometric analysis from three independent experiments shows a statistical significant difference between the levels of the H subunit between controls and DFP-treated mice in the pellet (B). By the colorimetric ferrozine method, a decrease in the levels of non-heme iron in the liver of DFP-treated FTL-Tg mice compared with non-treated FTL-Tg controls was observed in the supernatant, although it did not reach statistical significance (C).

Pathologic analysis was performed in tissue sections as described above. A remarkable difference from untreated FTL-Tg mice was the presence of a diffuse ferritin staining in tubule cells of the kidney, most remarkably in the DFP_100_ treated group (Fig 7A–7C). Histochemical analysis by Perls’ Prussian blue method showed the presence of iron in ferritin aggregates in the spleen of control FTL-Tg mice, which was clearly reduced in the DFP-treated group (Fig 7D–7F). To quantitatively characterize the impact of the chelator treatment on systemic ferritin deposition, we analyzed kidney protein samples by western blot analysis. The levels of the L, Lm, and H subunits in the supernatant were found significantly decreased in the DFP_100_ treated group (L, p = 0.0300; Lm, p = 0.0014; H, p = 0.0005). A significant decrease was also observed in the DFP_50_ treated group for the Lm and H subunits (Lm, p = 0.0248; H, p = 0.0124) (Fig 7G). A significant decrease was also observed in the levels of the L (p = 0.0035), Lm (p = 0.0009), and H (p = 0.0014) subunits in the pellet of the DFP_100_ treated group, but not in the DFP_50_ treated group (Fig 7H). A statistically significant decrease in the levels of non-heme iron in the kidney of DFP-treated FTL-Tg mice compared with non-treated FTL-Tg controls was observed by the colorimetric ferrozine method in the supernatant (Fig 7I) and in the pellet (Fig 7J) of the DFP_100_ group (p = 0.0299 and p = 0.0032), and in the pellet of the DFP_50_ group (p = 0.0190). A decrease was observed in the pellet of the DFP_50_ group, but did not reach statistical significance. Analysis of protein samples from the liver showed that the levels of the L and H subunits were not significantly changed in the supernatant of DFP-treated FTL-Tg mice. A decrease in the H levels was noted but failed to reach statistical significance (Fig 8A). In contrast, a significant decrease was observed in the levels of the H subunit in the pellet (DFP_50_; p = 0.092, DFP_100_; p = 0.0124) (Fig 8B). A decrease in the levels of non-heme iron in the liver of DFP-treated FTL-Tg mice was observed by the colorimetric ferrozine method in both supernatant and pellet, but it did not reach significance (Fig 8C). Analysis by multiplex RT-PCR of DFP-treated mice showed a significant decrease in the liver *mRNA* levels of *Ftl* (DFP_50_; p = 0.0049, DFP_100_; p = 0.0199) and *Fth1* (DFP_50_; p = 0.0006, DFP_100_; p = 0.0007), without significant changes in *Hamp* or *Tfrc mRNA* levels. A significant decrease in the *mRNA* levels of *Tfr2* (DFP_50_; p = 0.0075, DFP_100_; p = 0.0104), *Tf* (DFP_100_; p = 0.0146), *Aco1* (DFP_50_; p = 0.0003, DFP_100_; p = 0.0006), *Cp* (DFP_50_; p = 0.0072, DFP_100_; p = 0.0001), *Sod1* (DFP_50_; p = 0.0012, DFP_100_; p = 0.0010), *Sod2* (DFP_50_; p = 0.0003, DFP_100_; p = 0.0006), and *Homx2* (DFP_50_; p = 0.0153, DFP_100_; p = 0.0207) was observed. No significant changes were observed in the *mRNA* levels of *Hmox1* and additional genes analyzed in the liver plex (S3 Fig).

### Effects of the iron chelator DFP in brain iron homeostasis of FTL-Tg mice

**Fig 9:**
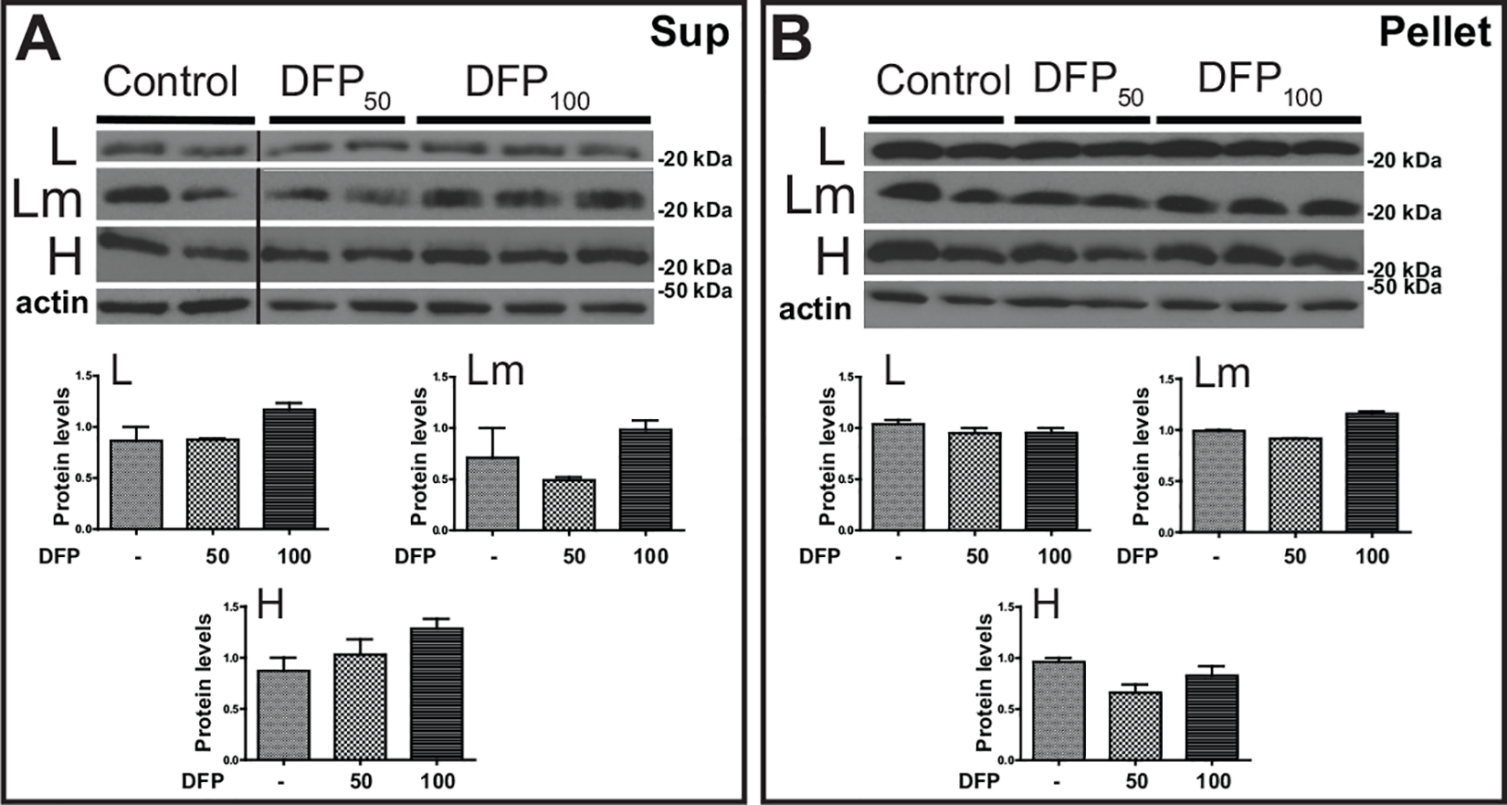
Western blot analysis of cerebral cortex of DFP-treated and control FTL-Tg mice. The levels of wild type L, Lm, and H polypeptides in the cerebral cortex in the supernatant (A) and the pellet (B) were determined by western blot. β-actin was used as loading control. The vertical lines in panel A denote non-adjacent bands from the same blot. Representative blots are shown for two control, two DFP_50_, and three DFP_100_ male mice. Densitometric analysis from three independent experiments shows no statistical significant difference between the groups.

To assess the impact of an iron chelation therapy on ferritin deposition in the CNS of FTL-Tg mice, we analyzed brains of FTL-Tg mice injected with DFP or normal saline as described above. Neuropathologic examination of brain tissues did not reveal major histological differences between FTL-Tg control mice and DFP treated mice (not shown). Western blot analysis of protein samples from the cerebral cortex showed that the levels of the L, Lm, and H subunits were not significantly different between control and DFP-treated FTL-Tg mice in both the supernatant and the pellet (Fig 9). Analysis by multiplex RT-PCR showed a significant decrease in the *mRNA* levels of *Ftl* (DFP_100_; p = 0.0233), without significant changes in *Fth1*, *Cp*, *Tfrc*, *Tf*, and *Sod1* levels. An increase in the *mRNA* levels of *Tfr2* was observed, but only reached significance at the low dose (DFP_50_; p = 0.0025). Significant increases in the *mRNA* levels of *Aco1* (DFP_100_; p = 0.0437) and *Sod2* (DFP_50_; p = 0.0217, DFP_100_; p = 0.0066) were also observed. No significant changes were observed in the *mRNA* levels of additional genes analyzed in the brain plex (S4 Fig). Analysis of the expression of the ferritin transgene by multiplex PCR did not reveal any significant differences in the cerebral expression of the transgene between controls and DFP-treated FTL-Tg mice (not shown).

## Discussion

Iron is a metal that is required as a cofactor in many metabolic processes in the CNS, including oxidative phosphorylation, neurotransmitter production, nitric oxide metabolism, and oxygen transport. Dysregulation of iron metabolism has been well-documented in neurodegenerative diseases, in particular in the disease HF, in which abnormal iron metabolism plays a primary role in the pathogenesis of the disease [1]. Herein, we present our results on the biological consequences of modifying iron levels *in vitro* and *in vivo* using an established transgenic mouse model of HF [10].

Using iMEFs from FTL-Tg mice, we examined the cellular response of mutant-containing ferritin to iron loading and chelation. We had previously proposed that iron may lead to enhanced transcription/translation of ferritin *mRNAs* and overproduction of ferritin by the cells in response to a diminished iron-storage ability of ferritin that contains Lm polypeptides [4, 11]. In iMEFs from FTL-Tg mice we observed a significant intracellular accumulation of ferritin and iron and an increase in susceptibility to oxidative damage with reduced cell viability when cells were exposed to iron, while treatment of the cells with the chelator DFP led to a significant improvement in cell viability and a decrease in iron content. Our data are in agreement with previous data obtained using astrocytes from FTL-Tg mice and fibroblasts from HF patients after being challenged with iron and chelators [5, 19], and support the notion that deranged iron metabolism plays a primary role in the pathogenesis of HF.

To determine the consequences of increased systemic iron *in vivo* in HF, systemic iron overload was induced in FTL-Tg mice by i.p. injections of iron. Our data demonstrated four chief findings. First, iron overload led to a significant increase in serum iron (Fe (III) bound to serum transferrin) and in the hematologic indices HtC, MCV, MCH, RDW, Pt, and MPV, without significant changes in RBC and WBC counts, serum Hb values, and MCHC. Second, iron overload did not cause identifiable histological changes in the brain, but iron-containing ferritin aggregates were observed in the liver and spleen. Third, western blot analysis showed a significant increase in the levels of the L and H subunits in the brain, but only in the insoluble fraction of iron overload mice. A significant change in levels of the L and H subunits, together with an increase in non-heme iron, was observed in the liver. Fourth, significant changes in *mRNA* levels compatible with iron overload were observed in the liver of iron overload mice, but these changes were not seen in the brain, where only the *mRNA* levels of *Tfrc* were significantly decreased. Iron overload led to a significant increase in liver hepcidin *mRNA* levels, which is produced predominantly by hepatocytes, as a response to an increase in serum iron levels. The increase may also reflect a response to an increase in liver iron levels or a mix of both [22].

A significant outcome from the iron overload study was that the overall impact of systemic iron overload in the CNS was not as noticeable as in the systemic compartment. The relative independence of the brain from iron in the systemic compartment may have protected the brain from acute changes in systemic iron [23]. Our data suggest that a small variation in iron body levels (from diet or other sources) may not significantly enhance CNS-related symptoms in patients with HF; however, changes in systemic iron levels may increase systemic ferritin deposition and lead to organ dysfunction. Since iron overload has a significant effect in systemic iron metabolism, as seen here in the liver and spleen of FTL-Tg mice, additional studies are needed to determine whether patients with HF may be more susceptible to hepatotoxicity and spleen dysfunction, the most common pathological findings in patients with iron overload [22]. Various systemic diseases have been reported in individuals affected by HF, often before the onset of neurological symptoms. These diseases include hypertension, diabetes mellitus, thrombosis, dyslipidemia, hepatitis, and chronic renal failure [3]. Whether these conditions are associated with the systemic deposition of ferritin in the organs involved awaits further investigation.

Iron chelation was induced in FTL-Tg mice by injecting the membrane permeable bidentate chelator DFP. DFP has been used clinically to treat iron overload, particularly hemosiderosis, and in experimental models. Despite its side effects, it currently represents the only possibility for removing and/or preventing iron accumulation in the brain [24, 25]. Some preliminary studies suggest that DFP may be effective in the management of neurological manifestations linked with iron accumulation [26]. The chelator forms a stable 3:1 chelant:iron(III) complex and has been reported to be able to cross the blood brain barrier (BBB) [25]. Total iron balance studies in iron overloaded thalassemia patients suggest that a dosage of 75 mg/kg/day of DFP may be comparable to a dosage of 40 mg/kg/day of desferoxamine [24]. In this study, DFP was administered i.p. because similar iron excretion is seen whether administered orally or i.p. [14]. Compared to controls, no significant changes in the hematological parameters were observed in animals in the low-dose group, but treatment with DFP at high dose led to a significant difference in the values of MCV, MCH, RDW, and MPV. Interestingly, it has been observed in iron overloaded thalassemia patients that a dose of 100 mg/kg/day of DFP increased the proportion of patients achieving negative iron balance [24].

DFP-treated FTL-Tg mice showed noteworthy histological and biochemical changes in the spleen and kidney, and significant changes in mRNA levels in the liver. Our data suggest that DFP treatment led to a reduction in ferritin and iron deposition in systemic organs such as kidney, spleen and liver, most remarkably in the high dose (DFP_100_) group. DFP may chelate iron from hepatocytes and the reticuloendothelial system in significant quantities, suggesting that chelators may be useful to treat systemic accumulation of ferritin in HF; however, increasing the dose of DFP to improve iron removal runs the risk of increasing the toxicity of the chelator. Although no animals were lost during the treatment, we observed some signs of toxicity in the DFP_100_ group, as it has been previously reported for DFP at high doses, with atrophy of the thymus, lymphoid tissues, and testis, and hypertrophy of the adrenals at doses of 100 mg/kg/day or greater in non-iron-loaded animals [16, 17]. Unfortunately, systemic ferritin deposits may not be useful to monitor therapeutic approaches since systemic deposits may be modified independently from brain ferritin deposits. While we observed significant systemic changes in iron metabolism and ferritin deposition, treatment with DFP (low and high dose) did not lead to noteworthy histological, biochemical, or gene expression changes in the brain of iron-chelated FTL-Tg mice. Our data is in agreement with previous observations indicating lack of effectivity of DFP in individuals affected by HF [2. 9]. Chinnery and col. [2] treated three patients with monthly venesection for 6 months. Two of the patients also were treated with intravenous desferrioxamine (4,000 mg weekly subcutaneously for up to 14 months), and one had oral DFP (2 g, three times a day for 2 months). These treatments caused profound and refractory iron depletion without significant benefits for the patients. Kubota and col. [9] treated a patient with monthly venesections (400 mL/mo) for 2 months without any changes in the clinical condition of the patient. In contrast, a recent study using a new mouse model of HF, expressing the same mutant form of FTL as the FTL-Tg mouse model, reported that oral treatment with DFP for 3 weeks reduced serum iron and the number and sizes of the iron positive granules in the brain [27]. The difference between this study and the present work may be due to i) the use of different promoters (the phosphoglycerate kinase (PGK) promoter [27] vs the mouse prion protein promoter [10] in this study) driving expression of the transgene, ii) the different ages of the mice (12 months vs 5–6 months in this study, to allow the detection of small changes in ferritin accumulation by changes in iron levels), iii) the number of mice used for the study (3 mice vs 11–14 mice per group in the present study to decrease the intrinsic variability between mice), and iv) the different approach to quantification.

In summary, our studies suggest that increases in systemic iron levels in patients with HF should not be expected to be markedly detrimental to the CNS pathology but may have a profound effect in systemic ferritin deposition, leading to a more significant manifestation of the systemic aspect(s) of the disease. We also demonstrated the usefulness of DFP to remove and redistribute iron and to resolubilize or prevent ferritin aggregation in systemic deposits. Further studies are needed to identify chelators with better BBB penetration or that may be delivered locally in the brain. The later may lead to lower toxicity by not significantly affecting systemic iron homeostasis.

## Supporting Information

S1 Fig Multiplex RT-PCR expression analysis of iron metabolism related genes in the liver of iron-loaded FTL-Tg mice. Bar graphs depict differential gene expression levels between controls and iron-loaded mice. Analysis was performed in triplicate and normalized to the *β-actin* gene. The group averages are reported as relative *mRNA* levels mean ± SD. Differences in gene expression were determined by two-tailed *t*-test.
(EPS)

S2 Fig Testicular atrophy in FTL-Tg mice treated with DFP at high dose (DFP_100_). Atrophy of the testes was noted at necropsy in the DFP_100_-treated group (A). A significant difference in weight of the testes was observed between control and DFP_100_-treated mice (p = 0.0013) (B).
(EPS)

S3 Fig Multiplex RT-PCR expression analysis of iron metabolism related genes in the liver of DFP-treated mice. Bar graphs depict differential gene expression levels between controls and DFP-treated FTL-Tg mice. Analysis was performed in triplicate and normalized to the *β-actin* gene. The group averages are reported as relative mRNA levels mean ± SD. Differences in gene expression were determined by two-tailed *t*-test.
(EPS)

S4 Fig Multiplex RT-PCR expression analysis of iron metabolism related genes in the cerebral cortex of DFP-treated mice. Bar graphs depict differential gene expression levels between control and DFP-treated FTL-Tg mice. Analysis was performed in triplicate and normalized to the Polr2a gene. The group averages are reported as relative mRNA levels mean ± SD. Differences in gene expression were determined by two-tailed t-test.
(EPS)
